# Don’t Be Afraid to Fail Because You Can Learn From It! How Intrinsic Motivation Leads to Enhanced Self-Development and Benevolent Leadership as a Boundary Condition

**DOI:** 10.3389/fpsyg.2020.00699

**Published:** 2020-04-16

**Authors:** Qiwei Zhou, Jih-Yu Mao, Fangcheng Tang

**Affiliations:** ^1^School of Economics and Management, Beijing University of Chemical Technology, Beijing, China; ^2^School of Business Administration, Southwestern University of Finance and Economics, Chengdu, China

**Keywords:** learning from failures, intrinsic motivation, self-development, benevolent leadership, self-determination

## Abstract

Employee learning from failures is key to effective employee functioning and long-term sustainable development. Although failure is an essential part of the learning process, it is less certain of why individuals would learn from failures and the benefits associated with it. Thus, it is significant to explore the cause and the consequence of learning from failures. Drawing upon self-determination theory, we explore the antecedent, consequence, and boundary condition of employee learning from failures. Random full-time employees in China were recruited to participate in the two-wave survey study via an online survey platform. Empirical results of 381 employees indicate that employee intrinsic motivation is positively related to employee learning from failures, which in turn facilitates employee self-development. Moreover, we found that benevolent leadership, a leadership style that is prevalent in the Chinese work context, plays an important moderating role in affecting the saliency of the indirect effect. Specifically, the indirect effect is more salient when benevolent leadership is higher. We test the hypotheses in SPSS using linear regressions and the PROCESS macro. Our study provides important implications for both theory and practice. Limitations and future research directions are also discussed.

“Even if I failed, the marks failures carved in my life would be a mark of glory. No matter where I go in the future, I will continue to fight like a warrior full of sportsman spirit. No matter what the situation is, I always know how to start afresh.”

— Lee and Lee (2012, p. 14)

## Introduction

On June 13, 2019, Lee Chong Wei, the formerly number-one-ranked and renowned Malaysian badminton player, tearfully announced his retirement, thereby ending his stellar 19-year playing career. Despite the fact that Lee Chong Wei has accumulated countless glories in international competitions, he has also failed in many important matches. The above quote in his autobiography was not only a prime illustration of his will and resilience for the game, but also how he self-developed into a better and more experienced player after each fall.

Since failures are prevalent in everyday work and the benefits of failures could be leveraged by making good use of them, learning from failures is of great importance to employee development. Many studies have confirmed on the positive influences of learning from failures. For example, learning from failures not only can enhance problem solving (Tucker and Edmondson, 2003) and future decision-making quality (Chuang and Baum, 2003), but also can reduce the likelihood of subsequent failures (Ingram and Baum, 1997; Kim et al., 2009) and enhance innovative performance (Homsma et al., 2009). Collectively, learning from failures improves performances at the individual- (e.g., Diwas et al., 2013), team- (e.g., Cannon and Edmondson, 2001), and organization-level (e.g., Zollo, 2009).

Although researchers have emphasized the theoretical and practical implications of employee learning from failures (e.g., Sitkin, 1992; Cannon and Edmondson, 2005; Argote and Miron-Spektor, 2011; Goodman et al., 2011; Chen et al., 2017; Dahlin et al., 2018), acknowledging failures may damage one’s reputation and image and elicit emotional burden, thus discouraging employees to admit and reflect upon failures (Dahlin et al., 2018). For example, employees may be reluctant to admit their mistakes in order to preserve face (e.g., Cannon and Edmondson, 2005; Argote and Miron-Spektor, 2011), and therefore may attribute their mistakes to external influences in order to avoid accountabilities. Also, a supportive and friendly work environment is critical to learning from failures because unfavorable stimuli such as bullying, hostility toward mistakes, and work-related stress can all discourage employees from recognizing their mistakes and hence reduce willingness to learn from failures (e.g., Eskildsen et al., 2015, 2016; Goodboy et al., 2017; Finstad et al., 2019). Consequently, this raises an interesting question that why are employees inclined to learn from failures and what is the underlying rationale.

Furthermore, learning from failures is critical to individuals’ future development. This is because learning from failures not only can help employees identify the gap between their actual capabilities and external environmental requirements, but also can provide them with lessons and experience to deal with problems in the future (Chen et al., 2017). Self-development indicates an employee’s self-motivated tendency to achieve progress by seeking and utilizing feedbacks, setting developmental goals, and engaging in related actions (London et al., 1999). While providing support for employee development is important in today’s dynamic business environment, it is also very important for employees themselves to self-initiate and engage in self-development. This is because individuals tend to be more strongly motivated by self-initiated developmental activities, and such self-driven developmental activities can also save training costs for organizations (Ellinger, 2004; Blau et al., 2008). Thus, unpacking the potential relationship between learning from failures and self-development is of great practical importance.

**FIGURE 1:**
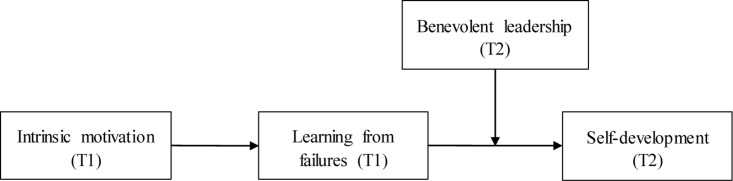
The overall studied model.

According to self-determination theory (SDT; Deci and Ryan, 1985, 1991, 2001), an employee’s level of devotion and dedication to work is fundamentally determined by his/her intrinsic motivation. Hence, in this study, we focus on employee learning from failures, and explore how intrinsic motivation can stimulate learning from failures, which in turn, enhance self-development through the lens of SDT. In addition, although existing literature has suggested that employee learning from failures can contribute to organizational functioning and efficiency, little is known about how leaders can help strengthen the effectiveness of employee learning (Cannon and Edmondson, 2005; Nembhard and Edmondson, 2006; Hirak et al., 2012). Leaders showing concerns and encouraging employees to overcome obstacles have been suggested to foster an individual’s self-determination process and development (e.g., Deci et al., 1989). By offering coaching, support and empathizing with employees, benevolent leadership shows individualized concerns for employees and facilitates their development (Cheng et al., 2000; Farh et al., 2008). Following this vein, we investigate the effect of benevolent leadership in cultivating the mediation effect of employee intrinsic motivation on self-development via learning from failures. Our research intends to contribute to the experiential learning literature by: (a) highlighting the significance of intrinsic motivation as an antecedent of employee learning from failures; (b) examining how learning from failures contributes to self-development; and (c) interrogating the boundary condition to which the effect of learning from failures can be strengthened. Figure 1 shows our overall theoretical model.

## Theory And Hypotheses

### Employee Learning From Failures and SDT

Employee learning from failures depicts the set of behaviors in which employees would undertake when facing problems at work. Specifically, employees not only would try to remedy current problems, but also would reflect on the underlying causes of the problems and take initiatives to make changes for the long-term benefit (Carmeli, 2007; Hirak et al., 2012). Learning from failures is a complex process and can be affected by many factors including individual and contextual influences, such as personalities (Zhao, 2011; Naveh et al., 2015), emotions (Tucker and Edmondson, 2003; Shepherd et al., 2011; Zhao, 2011), error management orientation (Rybowiak et al., 1999), leadership (Zhao, 2011; Hirak et al., 2012), and perceived organizational and leader support (Tucker and Edmondson, 2003; Edmondson, 2004).

Drawing upon SDT, this paper focuses on employee learning from failures, and examines its predictor, consequence, and boundary condition. SDT proposes that when employees perceive autonomy over workplace events, they would perceive their work as self-determining and would be more motivated to work, which would result in higher efficiency, more positive work attitudes, and better performance (Deci and Ryan, 2001; Pavot and Diener, 2013). Fulfilling one’s psychological needs (i.e., autonomy, relatedness, and competence) can help the individual to perceive work as self-determining. Notably, intrinsic motivation, which is a critical predictor of the self-determination process, has been found to promote employee learning-related activities at work, such as knowledge sharing (Hung et al., 2011) and knowledge transferring (Martín et al., 2009). Yet, the relationship between employee intrinsic motivation and learning from failures is rather underexplored. Thus, this paper proposes that employee intrinsic motivation is likely to facilitate learning from failures, which subsequently, promotes the individual’s self-development. In addition, according to Deci et al. (1989), leaders can also foster an environment for self-determination tendencies, where employees have higher trust, faith, and confidence, and thus exhibit more proactive behaviors. Based on which, the boundary condition of benevolent leadership is investigated.

### Employee Intrinsic Motivation and Learning From Failures

Dahlin et al. (2018) proposed that the motivation to allocate attention and cognitive resources serves as one of the important mechanisms leading employees to learn from failures. Studies have found that motivation to learn (Zhao, 2011) and safety motivation (Neal and Griffin, 2006) are both motivational factors that influence learning from failures. Among the various motivations, intrinsic motivation is one of the most important individual driving forces to affect an employee’s underlying goal and attitude toward exerting and sustaining effort in completing a job, thereby influencing an employee’s work experience and performance (Amabile et al., 1994).

Intrinsic motivation differs from extrinsic motivation in that intrinsically motivated individuals are driven to work because they enjoy the work itself, as opposed to by external rewards (Amabile et al., 1994). In other words, intrinsic motivation directly describes an employee’s perceived pleasure and satisfactory at work (Deci et al., 1989), and directly influences one’s dedication to work (Deci and Ryan, 1991; Deci et al., 1994). Intrinsically motivated employees strive to learn new skills and want to be deeply involved in tasks. They often consider their work as interesting, satisfying, and enjoyable, which lead to higher work engagement, performance, and creativity (Deci and Ryan, 1985, 2001; Amabile et al., 1994; Ryan and Deci, 2000).

Effective learning from failures not only requires employees to correctly attribute the underlying causes to failures, but also to further search for remedies or better solutions to prevent future errors (Dahlin et al., 2018). The positive relationship between intrinsic motivation and learning from failures is manifested in two aspects that reflect self-regulatory behaviors and discretionary efforts. First, intrinsically motivated employees tend to experience deeper pleasure of work (Deci and Ryan, 1985, 2001; Amabile et al., 1994; Ryan and Deci, 2000). They would be more active during work and can better grasp the meaning of work. Such employees are more involved in work and are more resilient when failure ensues. They are more willing to actively review and reflect on their past failed experiences and are more motivated to apply the experiences and lessons to future work. Second, intrinsically motivated employees are more willing to discuss their failures with others in order to receive feedback and make necessary adjustments going forward (Zhao, 2011), despite that failures can sometimes harm personal image and elicit psychological burdens (Dahlin et al., 2018). By sharing lessons with colleagues, employees can better reflect on their mistakes and accumulate wisdom from interpersonal interactions. Hence, we propose the following hypothesis:

Hypothesis 1: Employee intrinsic motivation is positively related to employee learning from failures.

### Employee Learning From Failures and Self-Development

Self-development reflects the extent to which an employee plans to be involved in tasks and acquire information and knowledge about how to sustain and improve performance in the long-term (London et al., 1999). Self-development is self-motivated and future-oriented. It captures behaviors such as seeking and utilizing feedback, setting long-term developmental goals, voluntarily participating in development-oriented activities, and actively paying attention to their progress (Williams et al., 1991; London et al., 1999), all of which are conducive to reducing organizational training cost (Ellinger, 2004) and enhancing employee commitment and satisfaction (Blau et al., 2008).

Studies have shown that learning-related environmental factors, such as learning climate, empowering climate, and feedback can impact employee self-development (e.g., London et al., 1999). We posit that employee learning from failures is positively related to employee self-development because learning from failures enhances one’s knowledge and stimulates positive attitude for long-term improvement. From a knowledge perspective, deep thinking produces more extensive and useful information (Morris and Moore, 2000), which help people accumulate experience and make more progress. By reviewing and reflecting on failed experiences, employees would identify the shortcomings of existing schemes (Sitkin, 1992; Baumard and Starbuck, 2005; Naveh et al., 2015). This helps them to evaluate current situations more accurately, develop a better understanding of their own weaknesses and shortcomings (Madsen and Desai, 2010), and identify the gaps between environmental requirements and personal abilities. Thus, employees are likely to take initiatives in acquiring appropriate skills and knowledge and making improvements in their future work. From an attitudinal perspective, failures evoke employees’ desire for future success (Chen et al., 2017) by promoting them to improve the *status quo* and engage in more developmental activities. Employees who learn from failures are more likely to set their own development goals and are more willing to pursue positive results. These arguments lead to our next hypothesis:

Hypothesis 2: Employee learning from failures is positively related to employee self-development.

Consistent with SDT, when seeking growth and progress are driven by intrinsic motivation, employees tend to take responsibility for their future development. They are likely to evaluate the gaps regarding personal abilities and environmental needs, prepare themselves with the relevant skills and knowledge to meet future demands, and actively seek feedback and set development-oriented goals (Williams et al., 1991; London et al., 1999; Blau et al., 2008). Hence, we expect a positive relationship between employee intrinsic motivation and self-development through learning from failures.

Intrinsically motivated employees are self-starters at work. They are more active and participative, and are more willing to review and reflect on failed experiences (Zhao, 2011). As such, learning from failures enriches employees’ knowledge pool and serves as a driving force to participate in development-oriented activities that help them to set personal goals. Therefore, the knowledge gained from failed experiences is likely to transfer employees’ intrinsic motivation into actions to achieve long-term development. Consequently, we hypothesize:

Hypothesis 3: Employee learning from failures mediates the relationship between employee intrinsic motivation and self-development.

### The Moderating Role of Benevolent Leadership

According to SDT, leaders also play an important role in fulfilling employees’ psychological needs and cultivating a self-deterministic environment (Van den Broeck et al., 2016). For instance, research has shown that leader inclusiveness (Hirak et al., 2012) and coaching behavior (Edmondson, 1999, 2004) would facilitate employee learning from failures. When granted with more autonomy from leaders, employees are likely to perceive a self-determining environment and be more confident, satisfied, and proactive at work (Deci et al., 1989; Gagné and Deci, 2005). Specifically, Deci et al. (1989) showed that leaders can help build this self-determining environment in three ways. First, leaders can empower employees to let them feel that they have more autonomy in decision making. Second, leaders can provide informative and constructive feedback to help employees feel competent in accomplishing job tasks. Also, leaders can show empathy to foster employees’ feelings of emotional connection with their leaders. All these behaviors illustrate a benevolent leadership style and are effective in enhancing employees’ motivation and willingness to work.

Benevolent leadership illustrates behaviors in which leaders show individualized care for employees within and out of work-related domains, such as tolerating and giving employees opportunities to correct mistakes, relieving their public embarrassment, providing coaching and mentoring, and demonstrating concerns for employees’ professional career path (Cheng et al., 2000; Farh et al., 2008). Benevolent leadership has been found to enhance employee satisfaction with the leader, organizational commitment, performance, and organizational citizenship behavior (Farh et al., 2008; Wang and Cheng, 2010). Such leadership elicits an impression that the leader is open, kind, approachable, and respects employees’ contributions.

Benevolent leadership is expected to strengthen the relationship between learning from failures and self-development. When leaders show benevolent behaviors, employees are inclined to build trust and harmonious interpersonal relationships with their leaders. Drawing upon SDT, benevolent leadership satisfies employees’ need for relatedness (Ryan and Deci, 2000), which in turn, strengthens feelings of self-determination. Benevolent leaders’ coaching behaviors encourage employees to set development goals, engage in developmental activities, and pay more attention to the impact of the progress they make (Farh et al., 2008). Hence, employees are more likely to acknowledge and be more resilient to failures, which facilitate them to self-initiate solutions and self-develop. At the same time, benevolent leadership nourishes an environment for open communication, mutual sharing, and active inquiry. This promotes employees to actively seek feedback and make improvements, which facilitate their self-development (Wang and Cheng, 2010).

Moreover, benevolent leadership characterizes interpersonal caring (Cheng et al., 2000). When employees encounter failure, benevolent leaders are less likely to solely evaluate the incidence based on the outcome, but rather, are inclined to focus on the process and help employees reflect on the error. Benevolent leaders are also willing to help employees overcome fears and disappointments through communication and support, and inspire them to establish development-oriented goals in the long-term (Farh et al., 2008). Their individualized care can alleviate employees’ negative emotions derived from failed experiences and help them to apply failed experiences into future development. On the contrary, lack of benevolence makes employees feel that their leaders are insensitive about their feelings and are intolerant and critical of their mistakes. This elicits psychological burden in employees and inhibits their motivation to apply failed experiences to future work. In this circumstance, employees tend to find linking personal growth to organizational development difficult, and thus, the motivations to engage in development-related activities and contribute to organizational functioning are likely to be diminished. Taken together, we propose the following moderation hypothesis:

Hypothesis 4: Benevolent leadership moderates the relationship between employee learning from failures and self-development, such that the relationship is stronger when benevolent leadership is high rather than low.

### An Integrated Model

Drawing upon SDT, we predict that intrinsically motivated employees are likely to acknowledge and reflect on failed experiences, which stimulate them to learn from their failures. This, in turn, helps them to reach higher self-development. In addition, when leaders show benevolence toward them, they are more likely to feel an obligation to repay the leader and organization. Hence, they are inclined to translate failed experiences into means of becoming a more developed employee that can better contribute to the organization in the long-run. As a result, we expect that the indirect effect of employee intrinsic motivation on self-development through learning from failures is likely to be strengthened when benevolent leadership is higher. Therefore, we postulate the following integrated hypothesis:

Hypothesis 5: Benevolent leadership moderates the indirect effect of employee intrinsic motivation on self-development through learning from failures, such that this indirect effect is stronger when benevolent leadership is high rather than low.

## Method

### Participants and Procedures

We designed a multi-time (i.e., Time 1 and Time 2) survey study to test our hypotheses. We distributed questionnaires via an online survey platform, Sojump (wjx.cn), to random employees who had registered on the platform around China. Given the budget constraint and the expected survey response rate, we paid and asked the survey platform to recruit 800 potential participants over a period of 3 days. The inclusion criteria were if the participants were working as a full-time employee and had a familiar direct supervisor to report to. Participation in the study was based on voluntariness and participants were compensated upon completion of each survey questionnaire. We told our participants that their responses would be kept confidential and only be used for research purpose.

In the Time 1 survey, we asked our participants to assess their intrinsic motivation and learning from failures as well as provide their demographic information. In one week, we received responses from 663 participants (response rate = 82.9%). Two weeks later, at Time 2, we asked the 663 participants to evaluate their self-development and their direct supervisor’s benevolent leadership. In one week, we received responses from 475 participants (response rate = 71.6%). A major reason for the loss of samples in between the data collections was perhaps due to the study design which was conducted online as opposed to in person. Nevertheless, the loss of samples in between the data collections was acceptable, as the response rates were comparable to other organizational studies that had also adopted a 2-week time lag in between data collections (e.g., Kabat-Farr et al., 2019). After eliminating incomplete responses and matching surveys at both times with a unique identification code, we obtained a final sample of 381 employees.

Among the participants, 56.4% were female and their age mainly ranged from 26 to 35 (52.7%). Most of them had obtained a bachelor degree (69.6%). Their job positions were as follows: 29.9% were frontline employees; 39.6% were junior managers; and 27.6% were middle-level managers. The average tenure with their direct supervisor was 3.72 years [standard deviation (*SD*) = 2.88].

### Measures

As all administered items were in Chinese, we followed Brislin’s (1986) translation and back-translation procedures to ensure the quality of the translated survey items. All items were assessed on a 7-point scale ranging from 1 (strongly disagree) to 7 (strongly agree).

#### Intrinsic Motivation

Employees evaluated their intrinsic motivation using the short 6-item scale adapted from the Work Preference Inventory (Amabile et al., 1994; Tremblay et al., 2009). An example item featured “I enjoy doing work that is so absorbing that I forget about everything else” [Cronbach’s alpha (α) = 0.78].

#### Learning From Failures

Employees assessed their learning from failures on the 5-item scale administered by Hirak et al. (2012). A sample item was “When I make a mistake, I inform the relevant supervisor to enable others to learn from it” (α = 0.70).

#### Benevolent Leadership

Employees rated their direct supervisor’s benevolent leadership using Cheng et al. (2000) 11-item scale. One item was dropped due to the low factor loading. An example item was “My supervisor will help me when I am in an emergency” (α = 0.84).

#### Self-Development

Self-development was assessed using the four items from London et al. (1999). A featured item was “I have committed myself to improve my job performance in the future” (α = 0.70).

#### Control Variables

Several demographic variables were included as control variables. We controlled for employee gender (0 = female; 1 = male), age (1 = 20 years old or younger; 2 = 21–25 years old; 3 = 26–30 years old; 4 = 31–35 years old; 5 = 36–40 years old; 6 = 41–45 years old; 7 = 46–50 years old; 8 = 51–55 years old; 9 = 56–60 years old; 10 = above 60 years old), education (1 = vocational school/technical school; 2 = high school; 3 = junior college; 4 = bachelor; 5 = master; 6 = doctorate), job position (1 = frontline employee; 2 = junior manager; 3 = senior manager; 4 = top manager), and tenure with direct supervisor (in years) because these demographic variables have been argued to affect perceptions of social interactions and behavioral outcomes (e.g., Ng and Feldman, 2010).

### Analytical Strategy

First, to examine the empirical distinctiveness of the focal constructs, we used Lisrel 8.8 to perform confirmatory factor analysis with all items as indicators. Second, we tested the extent to which our findings are affected by common method variance (Podsakoff et al., 2003) by performing the Harman’s single-factor test (Podsakoff and Organ, 1986). Third, we used SPSS 22.0 to conduct linear regressions to test the direct effects (i.e., Hypotheses 1 and 2). We used Model 4 in SPSS PROCESS macro (Hayes, 2013) to test the mediation effect (i.e., Hypothesis 3), Model 1 to test the moderation effect (i.e., Hypothesis 4), and Model 14 to test the conditional indirect effect (i.e., Hypothesis 5).

## Results

### Confirmatory Factor Analysis

We used Lisrel 8.8 to conduct confirmatory factor analysis to examine the empirical distinctiveness of our main constructs. As shown in Table 1, results suggest that the hypothesized four-factor model demonstrates good fit [χ*^2^*(266) = 471.61, *p* < 0.001; SRMR = 0.05, TLI = 0.90, CFI = 0.92, RMSEA = 0.05; Kline, 2010], and provides significant improvement in the chi-square value over all other alternative models. Thus, the main constructs are statistically different from one another.

**TABLE 1:** Confirmatory factor analysis results.

Models	χ^2^	*df*	Δχ^2^ (Δ*df*)	RMSEA	SRMR	CFI	TLI
**Four-factor model**							
The hypothesized four-factor model	471.61^∗∗∗^	266	—	0.05	0.05	0.92	0.90
**Three-factor model**							
Combining learning from failures and self-development	591.35^∗∗∗^	269	119.74 (3)^∗∗∗^	0.06	0.06	0.88	0.86
Combining intrinsic motivation and learning from failures	610.65^∗∗∗^	269	139.04 (3)^∗∗∗^	0.06	0.07	0.87	0.85
Combining benevolent leadership and learning from failures	652.41^∗∗∗^	269	180.80 (3)^∗∗∗^	0.06	0.08	0.85	0.84
**Two-factor model**							
Combining benevolent leadership, learning from failures, and self-development	855.62^∗∗∗^	271	384.01 (5)^∗∗∗^	0.08	0.08	0.80	0.77
Combining intrinsic motivation and benevolent leadership; combining learning from failures and self-development	1326.05^∗∗∗^	271	854.44 (5)^∗∗∗^	0.10	0.09	0.68	0.65
**One-factor model**							
Combining all variables	1579.95^∗∗∗^	272	1108.34 (6)^∗∗∗^	0.11	0.10	0.62	0.58

Δχ^2^*is derived from comparisons with the hypothesized four-factor model. χ^2^, chi-square; df, degrees of freedom; RMSEA, root mean square error of approximation; SRMR, standardized root mean square residual; CFI, comparative fit index; TLI, Tucker-Lewis index.^∗∗∗^p < 0.001.*

### Test of Common Method Bias

To assess the extent to which our findings are affected by common method variance (Podsakoff et al., 2003), we conducted Harman’s single-factor test (Podsakoff and Organ, 1986). The variance explained by the first factor from explanatory factor analysis is 19.41%, which is lower than the 50% threshold (Hair et al., 1998). In addition, the variance inflation factor for all variables is lower than 10. Furthermore, as shown in Table 1, the hypothesized four-factor model has superior model fit indices than the alternative one-factor model [χ*^2^*(272) = 1579.95, *p* < 0.001; SRMR = 0.10, TLI = 0.58, CFI = 0.62, RMSEA = 0.11]. Thus, common method variance and multicollinearity issues did not substantially affect our findings.

### Hypothesis Tests

Table 2 presents the means, standard deviations, inter-correlations, and reliabilities of the studied variables. We used SPSS 22.0 to conduct linear regressions to test our hypotheses. All control variables (i.e., gender, age, education, job position, tenure with supervisor) were included in the analyses.

**TABLE 2:** Means, standard deviations, correlations, and reliabilities among studied variables.

Variables	Mean	*SD*	1	2	3	4	5	6	7	8	9
1. Gender	−	0.50	−								
2. Age	−	1.58	0.11^∗^	−							
3. Education	−	0.68	0.04	–0.18^∗∗^	−						
4. Job position	−	0.83	0.29^∗∗^	0.42^∗∗^	0.21^∗∗^	−					
5. Tenure with supervisor	3.72	2.88	0.03	0.56^∗∗^	–0.07	0.35^∗∗^	−				
6. Intrinsic motivation (T1)	4.94	0.89	0.07	–0.02	–0.01	0.12^∗^	–0.03	(0.78)			
7. Learning from failures (T1)	5.21	0.73	0.08	0.18^∗∗^	–0.02	0.21^∗∗^	0.12^∗^	0.35^∗∗^	(0.70)		
8. Benevolent leadership (T2)	4.97	0.82	0.07	−0.11^∗^	0.09	0.09	–0.02	0.29^∗∗^	0.27^∗∗^	(0.84)	
9. Self-development (T2)	5.95	0.58	−0.11^∗^	–0.01	0.08	0.05	0.04	0.21^∗∗^	0.28^∗∗^	0.24^∗∗^	(0.70)

*N = 381. SD = standard deviation; T1 = data collected at Time 1; T2 = data collected at Time 2. Cronbach’s alphas are shown in parentheses along the diagonal. The coding and actual percentage of the categorical demographic variables are as follows: Gender: 0 = female (56.4%), 1 = male (43.6%). Age: 1 = 20 years old or younger (2.6%), 2 = 21–25 years old (16.8%), 3 = 26–30 years old (23.6%), 4 = 31–35 years old (29.1%), 5 = 36–40 years old (14.4%), 6 = 41–45 years old (5.2%), 7 = 46–50 years old (5.5%), 8 = 51–55 years old (2.2%), 9 = 56–60 years old (0.3%), 10 = above 60 years old (0.3%). Education: 1 = vocational school/technical secondary school (0.5%), 2 = high school (3.9%), 3 = junior college (11.8%), 4 = bachelor (69.6%), 5 = master (14.2%), 6 = doctorate (0%). Job position: 1 = frontline employee (29.9%), 2 = junior manager (39.6%), 3 = senior manager (27.6%), 4 = top manager (2.9%).^∗^p < 0.05, ^∗∗^p < 0.01, ^∗∗∗^p < 0.001.*

#### Test of Direct Effects

Hypothesis 1 posits that employee intrinsic motivation is positively related to employee learning from failures. Results displayed in Table 3 indicate that employee intrinsic motivation is positively associated with employee learning from failures [*b* = 0.27, standard error (*SE*) = 0.04, *p* < 0.001]. Thus, Hypothesis 1 is supported.

**TABLE 3:** Linear regression results.

Variables	Learning from failures	Self-development	
		
	*b* (*SE*)	*b* (*SE*)	*b* (*SE*)
Intercept	3.51^∗∗∗^ (0.31)	4.27^∗∗∗^ (0.29)	6.50^∗∗∗^ (1.28)
**Controls**			
Gender	0.02 (0.07)	−0.16^∗∗^ (0.06)	−0.16^∗∗^ (0.06)
Age	0.05 (0.03)	−0.02 (0.02)	−0.00 (0.02)
Education	−0.02 (0.05)	0.08 (0.05)	0.07 (0.04)
Job position	0.10 (0.05)	0.00 (0.04)	−0.01 (0.04)
Tenure with supervisor	0.01 (0.02)	0.01 (0.01)	0.01 (0.01)
**Independent variable**			
Employee intrinsic motivation	0.27^∗∗∗^ (0.04)	0.09^∗^ (0.03)	0.06 (0.03)
**Mediator**			
Learning from failures		0.20^∗∗∗^ (0.04)	−0.30 (0.24)
**Moderator**			
Benevolent leadership			−0.40 (0.25)
**Interactions**			
Learning from failures × Benevolent leadership			0.10^∗^ (0.05)
*R*^2^	0.16	0.12	0.15
Adjusted *R*^2^	0.15	0.11	0.13
*F*	12.20^∗∗∗^	7.56^∗∗∗^	7.38^∗∗∗^

*N = 381. Unstandardized regression coefficients (and standard errors) are reported. Gender: 0 = female, 1 = male. Age: 1 = 20 years old or younger, 2 = 21–25 years old, 3 = 26–30 years old, 4 = 31–35 years old, 5 = 36–40 years old, 6 = 41–45 years old, 7 = 46–50 years old, 8 = 51–55 years old, 9 = 56–60 years old, 10 = above 60 years old. Education: 1 = vocational school/technical secondary school, 2 = high school, 3 = junior college, 4 = bachelor, 5 = master, 6 = doctorate. Job position: 1 = frontline employee, 2 = junior manager, 3 = senior manager, 4 = top manager.^∗^p < 0.05, ^∗∗^p < 0.01, ^∗∗∗^p < 0.001.*

Hypothesis 2 argues that employee learning from failures is positively related to employee self-development. The results show that employee learning from failures is indeed positively associated with employee self-development (*b* = 0.20, *SE* = 0.04, *p* < 0.001). Hence, Hypothesis 2 receives support.

#### Test of Mediation Effect

Hypothesis 3 proposes that employee learning from failures mediates the relationship between intrinsic motivation and self-development. We used Model 4 in SPSS PROCESS macro (Hayes, 2013) to test this hypothesis. Results from bootstrapping analysis (with 5000 resamples) reveal a significant positive indirect effect [*indirect effect* = 0.05, *SE* = 0.01, *95% confidence interval* (*CI*) = [0.03, 0.09]]. Therefore, Hypothesis 3 is supported.

#### Test of Moderation Effect

**FIGURE 2:**
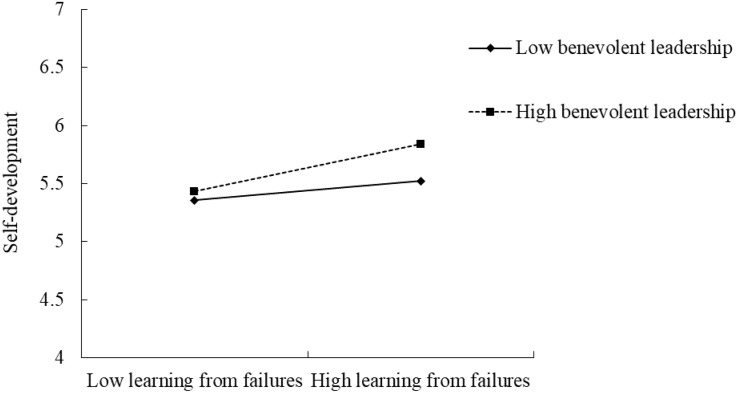
The interaction effect of learning from failures and benevolent leadership on self-development.

Hypothesis 4 predicts that benevolent leadership moderates the relationship between employee learning from failures and self-development, such that the relationship is stronger when benevolent leadership is high rather than low. We used Model 1 in SPSS PROCESS macro (Hayes, 2013) to test this hypothesis. Results shown in Table 3 reveal a significant interaction (*b* = 0.10, *SE* = 0.05, *p* < 0.05), and Figure 2 illustrates the interaction pattern (Aiken and West, 1991). Results from the simple slope test indicate that the positive effect of learning from failures on self-development is stronger when benevolent leadership is one *SD* above the mean (*simple slope* = 0.26, *SE* = 0.06, *t* = 4.35, *p* < 0.001) than when benevolent leadership is one *SD* below the mean (*simple slope* = 0.10, *SE* = 0.06, *t* = 1.86, *ns*). Thus, Hypothesis 4 is supported.

#### Test of Conditional Indirect Effect

Hypothesis 5 predicts that benevolent leadership moderates the indirect effect of employee intrinsic motivation on self-development through learning from failures, such that this indirect effect is stronger when benevolent leadership is high rather than low. We used Model 14 in SPSS PROCESS macro (Hayes, 2013) to test this hypothesis. Following Preacher et al. (2007) recommendations, we tested the conditional indirect effect by conducting a moderated path analysis using 5000 bootstrap resamples to construct 95% bias-corrected CIs for the indirect effect through learning from failures.

Results presented in Table 4 indicate that benevolent leadership moderates the indirect effect of intrinsic motivation on self-development through learning from failures (*index of moderated mediation* = 0.03, *SE* = 0.01, 95% CI = [0.007, 0.05]). Specifically, the indirect effect is stronger when benevolent leadership is one *SD* above the mean (*indirect effect* = 0.07, *SE* = 0.02, 95% CI = [0.04, 0.11]) than when benevolent leadership is one *SD* below the mean (*indirect effect* = 0.03, *SE* = 0.02, 95% CI = [0.002, 0.06]). Hence, Hypothesis 5 is supported.

**TABLE 4:** Conditional indirect effect results (bootstrapping method with 5000 resamples).

**Conditional indirect effects**				
**Moderator: Benevolent leadership**	**Effect**	**SE**	**Lower level CI**	**Upper level CI**
−1 SD (4.15)	0.03	0.02	0.002	0.06
+1 SD (5.80)	0.07	0.02	0.04	0.11
**Index of moderated mediation**				
**Index**	**SE**		**Lower level CI**	**Upper level CI**
0.03	0.01		0.007	0.05

*SD, standard deviation; SE, standard error; CI, confidence interval tested at 95% significance level.*

## Discussion

In today’s rapid changing environment, employees frequently encounter unexpected failures in the workplace. Investigating how to take advantage of failures has substantial implications on employees’ long-term development. Based on SDT, our study seeks to extend previous research on employee learning from failures by extending its antecedent, consequence, and boundary condition. Results show that employee intrinsic motivation positively predicts learning from failures; learning from failures promotes self-development; and learning from failures mediates the relationship between intrinsic motivation and self-development. Further, benevolent leadership strengthens the positive relationship between learning from failures and self-development and the indirect effect of intrinsic motivation on self-development via learning from failures. Our hypotheses remain supported when all control variables are excluded. These results suggest that our findings, to a large extent, are robust.

Since our samples were randomly recruited, our findings demonstrate a considerable extent of external validity. However, despite our findings, employees should always be attentive and mindful in job functioning and try to avoid making mistakes because erroneous procedures can be costly and render substantial impact, especially in sectors where the margin of error is thin. For instance, a bank teller making a numerical error on a bill can result in substantial loss for the bank. A pilot mistakenly turns off a switch can seal the fate of the aircraft. Hence, although learning from failures can help employees grow and mature, employees should always strive to refrain from making errors at work. If they do inevitably make mistakes, they should self-motivate to learn from them and avoid making the same mistakes in the future.

By pinpointing intrinsic motivation as an antecedent of employee learning from failures, we echo the contention that motivation is a critical driving force for deliberate learning (Dahlin et al., 2018), which has also been found in empirical studies for managers (Baumard and Starbuck, 2005) and entrepreneurs (Eggers and Song, 2015). Compared to previous research that had investigated motivation to learn (Zhao, 2011) and safety motivation (Neal and Griffin, 2006), we argued and found support for the important antecedent role of intrinsic motivation. Moreover, existing research has mainly focused on how learning from failures can enhance job performance, such as problem-solving quality (Chuang and Baum, 2003; Tucker and Edmondson, 2003), in-role performance (Diwas et al., 2013), innovative performance (Homsma et al., 2009), and reduced subsequent failure rate (Ingram and Baum, 1997; Kim et al., 2009). Our findings extend understanding of the consequences of learning from failures by also discovering a positive relationship between learning from failures and self-development. In addition, we also investigate the critical leadership role in affecting employee learning effectiveness. This echoes previous studies which have suggested that leader inclusiveness (Hirak et al., 2012), effective coaching (Cannon and Edmondson, 2001), and managerial opinions of errors (Zhao, 2011) can affect learning effectiveness. We found that employee self-development would be further amplified when leaders display benevolence that encourages employees to apply their failed experiences to benefit their work in future. We further interpret our findings, contributions, and limitations in the following sections.

### Theoretical Implications

Our findings contribute to theory and research in several ways. First, we contribute to the experiential learning literature by identifying intrinsic motivation as an important antecedent, in addition to other known antecedents such as personality and affectivity (Zhao, 2011) as well as other proximal failure-related orientations (Rybowiak et al., 1999; Shepherd et al., 2011). Previous research has argued that not every failed experience does indeed lead to effective learning (e.g., Tucker and Edmondson, 2003; Baumard and Starbuck, 2005). This suggests that even though failures present individuals learning opportunities, lack of learning motivation can prevent individuals from engaging in the actual learning. Thus, consistent with Dahlin et al.’s (2018) theoretical framework, we adopt SDT and associate learning from failures with a specific type of motivation, and highlight the important role of intrinsic motivation in promoting such learning behavior. Accordingly, we can draw conclusions that, despite the learning opportunities, intrinsic motivation serves as a prerequisite that activates individuals’ learning process.

Second, our findings broaden the understanding of how learning from failures can facilitate employee development. Existing studies on the outcomes of learning from failures are generally limited to its influence on performance (e.g., Sitkin, 1992; Cannon and Edmondson, 2001; Zhao, 2011; Hirak et al., 2012; Diwas et al., 2013), creativity (Homsma et al., 2009), and decreased errors (Edmondson, 2004; Baumard and Starbuck, 2005; Naveh et al., 2015). Our findings show that learning from failures encourages employees to self-develop, which not only has practical meanings but also enhances understanding of the antecedent of self-development and its underlying rationale. Although scholars have noted the importance of learning in facilitating self-development (e.g., London et al., 1999; Morris and Moore, 2000), they have mostly focused on general learning as opposed to linking learning to a specific context. Our model explicitly studies learning in the context of failed experiences and discovers its unique importance on self-development, which is beneficial to help employees confront and succeed in the complex and rapidly changing business environment.

Third, we further clarify the role of benevolent leadership in cultivating the relationship between learning from failures and self-development. Previous research has mostly studied the direct influence of benevolent leadership on employee outcomes (e.g., Chan and Mak, 2012), but has not paid much attention to its contingent influence on employees’ self-development process. Self-development is not only affected by individual factors, but can also be simultaneously impacted by leadership factors (Orvis and Leffler, 2011). Hence, instead of focusing on the direct influence of benevolent leadership on employee self-development, we adopt an interactionist approach and examine the interplay of learning from failures and benevolent leadership on employee self-development. In doing so, we found that caring and showing concerns for employees are likely to facilitate employees’ self-determination process, and thus encourage them to self-develop. By unpacking the contingent influence of benevolent leadership, we further enhance understanding of the critical role of leadership influence in shaping employee self-development in the workplace.

### Practical Implications

Our findings have multiple implications for managerial practices. First, focusing on learning from failures provides managers a clearer and more comprehensive framework of guiding and encouraging employees to learn from their own mistakes. Failures provide employees with valuable opportunities to learn, which can further lead to self-initiated development. Employees should not be deflated from failures and errors, but instead, should reflect upon and make the best use of them. Although many employees are well aware of the important role of learning from failures in facilitating individual development, many do not actually engage in such learning (Husted and Michailova, 2002). This is because learning from failures requires employees to engage in analytical thinking in order to make the necessary adjustments going forward. However, many employees are reluctant to do so because of lack of ownership and internalization of their job roles (Dawkins et al., 2017). Thus, it is important for managers to help employees internalize and take responsibility of their job roles. For example, managers can empower their employees more and let them participate more in decision making (Qian et al., 2018). Managers can also organize regular discussion seminars and ask employees to review and reflect on their jobs as well as provide feedback for each other. Managers should use effective leadership behaviors and implement practices to ensure their employees’ intrinsic motivation.

Second, it is virtually impossible for employees to not make any mistakes. Since failures often reduce one’s self-esteem, self-efficacy, and produce negative emotions (e.g., Zhao, 2011), it is important for managers to provide a supportive and caring environment. Even though emphasizing normative procedures, systems, and work standards can help employees realize their shortcomings in work, managers should be careful not to elicit feelings in which employees perceive that managers lack tolerance for failures and mistakes. As evident in our findings, benevolent leadership is a critical boundary condition of effective learning from failures. Because many uncertainties can arise during the learning process (Chen et al., 2017; Dahlin et al., 2018), exhibiting benevolence serves as an important and feasible mean to reduce help employees relieve psychological burden and strengthen their self-determinations. For instance, managers can approach employee failures from a caring and encouragement perspective. They can provide comfort and specific feedback to allow employees better reconstruct and reflect on the critical events leading up to the failures. Managers can also share their own failed experiences with employees to help them learn from their mistakes and develop.

### Limitations and Directions for Future Research

Although our study has many strengths, including the adoption of a multi-time survey design, there are several limitations worth noting. First, since our focal variables are reported by the same source (i.e., employee rated), our hypothesized relationships may be vulnerable to common method variance (Podsakoff et al., 2012). In order to alleviate this problem, we not only conducted Harman’s single-factor test, but also adopted a multi-time design (Podsakoff et al., 2012; Beal, 2015). At the same time, studies have shown that common method variance can inhibit moderation effects (Siemsen et al., 2010), which suggests that our moderation result can be underestimated. Hence, we encourage future research to collect data from multiple sources and incorporate samples from different contexts to enhance the generalizability of our findings.

Second, the Cronbach’s alphas for learning from failures and self-development are both 0.70. Although this satisfies the accepted standard (Nunnally, 1978), higher Cronbach’s alpha suggests that the measurement items have higher correlations and lower variance among them. However, this should not substantially affect the study results because the items were averaged prior to estimating the relationships among variables of interest. Moreover, since calculation of the Cronbach’s alpha is a function of the number of items, scales with fewer measurement items tend to yield lower Cronbach’s alphas. Nevertheless, we urge future research to adopt other measurement scales to further validate our findings.

Despite the limitations, our study sheds light on a few potential interesting research avenues. While we examined the mediating role of employee learning from failures between intrinsic motivation and self-development, it is worthwhile for future research to explore other mechanisms linking these two variables. As learning behavior likely involves enriched knowledge and modified working procedures (Walumbwa et al., 2009), cognitive appraisals and other attitudinal or affective factors may also play significant roles in facilitating the relationship and promoting employees to self-develop.

In addition, we explored a particular leadership behavior, namely benevolent leadership, as the moderating influence of learning from failures on self-development. We also urge future studies to explore other contextual influences. For instance, organizational and team influences are expected to affect the self-determination process in learning from failures. A climate that encourages team members to share knowledge with one another is likely to provide more diversified information and feedback to the members (Xue et al., 2011). As such, employees would have more diversified angles to view and reflect on their failed experiences. Future research is also encouraged to explore inhibitive influences that can decrease employees’ willingness to learn. For example, work-related stress not only consumes employees’ cognitive resources which hinder their ability to focus on job tasks, but also decreases their willingness to face and cope with negative events (Ganster and Rosen, 2013), thus reducing their motivation to learn from failures. Moreover, bullying and abusive behaviors can impede employee learning from failures. This is because employees can be less tentative to acknowledge their mistakes in order to avoid being ridiculed and harassed. Future studies can empirically investigate these negative influences on employee learning from failures, and explore employees’ coping strategies.

## Conclusion

Drawing upon SDT, we found that employee learning from failures is predicted by one’s intrinsic motivation and affects one’s subsequent self-development. Furthermore, we identified benevolent leadership as an important boundary condition. Specifically, our research contributes to literatures on experiential learning and self-determination by: (a) highlighting the importance of intrinsic motivation as an antecedent of employee learning from failures; (b) examining how learning from failures facilitates self-development; and (c) interrogating the boundary condition to which the positive effect of learning from failures can be strengthened. Altogether, this study highlights the importance of self-determination in the process of achieving better self-development and the valuable role for managers to facilitate this process.

## Data Availability Statement

The data used in this study are available upon request to the corresponding author.

## Ethics Statement

The study was reviewed and approved by the Ethics Committee of Tsinghua University. Informed consent was obtained from all participants.

## Author Contributions

QZ drafted the manuscript. J-YM edited the manuscript. FT provided theoretical guidance.

## Conflict of Interest

The authors declare that the research was conducted in the absence of any commercial or financial relationships that could be construed as a potential conflict of interest.
